# Neisseria meningitidis Induces Pathology-Associated Cellular and Molecular Changes in Trigeminal Schwann Cells

**DOI:** 10.1128/IAI.00955-19

**Published:** 2020-03-23

**Authors:** Ali Delbaz, Mo Chen, Freda E.-C. Jen, Benjamin L. Schulz, Alain-Dominique Gorse, Michael P. Jennings, James A. St John, Jenny A. K. Ekberg

**Affiliations:** a Clem Jones Centre for Neurobiology and Stem Cell Research, Griffith University, Brisbane, QLD, Australia; b Griffith Institute for Drug Discovery, Griffith University, Brisbane, QLD, Australia; c Menzies Health Institute Queensland, Griffith University, Southport, QLD, Australia; d Institute for Glycomics, Griffith University, Southport, QLD, Australia; e Australian Infectious Diseases Research Centre, School of Chemistry and Molecular Biosciences, the University of Queensland, St. Lucia, Brisbane, Australia; f QFAB Bioinformatics, Institute for Molecular Bioscience, The University of Queensland, St. Lucia, Brisbane, Australia; University of California, Davis

**Keywords:** Gram-negative bacteria, glial cell, multinucleated, cranial nerves, cancer, glioma, central nervous system infections, infection route, proteomics, trigeminal nerve, cranial nerve

## Abstract

Neisseria meningitidis, a common cause of sepsis and bacterial meningitis, infects the meninges and central nervous system (CNS), primarily via paracellular traversal across the blood-brain barrier (BBB) or blood-cerebrospinal fluid barrier. N. meningitidis is often present asymptomatically in the nasopharynx, and the nerves extending between the nasal cavity and the brain constitute an alternative route by which the meningococci may reach the CNS. To date, the cellular mechanisms involved in nerve infection are not fully understood. Peripheral nerve glial cells are phagocytic and are capable of eliminating microorganisms, but some pathogens may be able to overcome this protection mechanism and instead infect the glia, causing cell death or pathology. Here, we show that N. meningitidis readily infects trigeminal Schwann cells (the glial cells of the trigeminal nerve) *in vitro* in both two-dimensional and three-dimensional cell cultures. Infection of trigeminal Schwann cells may be one mechanism by which N. meningitidis is able to invade the CNS. Infection of the cells led to multinucleation and the appearance of atypical nuclei, with the presence of horseshoe nuclei and the budding of nuclei increasing over time. Using sequential window acquisition of all theoretical mass spectra (SWATH-MS) proteomics followed by bioinformatics pathway analysis, we showed that N. meningitidis induced protein alterations in the glia that were associated with altered intercellular signaling, cell-cell interactions, and cellular movement. The analysis also suggested that the alterations in protein levels were consistent with changes occurring in cancer. Thus, infection of the trigeminal nerve by N. meningitidis may have ongoing adverse effects on the biology of Schwann cells, which may lead to pathology.

## Abstract (precis)

Neisseria meningitidis, a common cause of sepsis and bacterial meningitis, infects the meninges and central nervous system (CNS), primarily via paracellular traversal across the blood-brain barrier (BBB) or blood-cerebrospinal fluid barrier. N. meningitidis is often present asymptomatically in the nasopharynx, and the nerves extending between the nasal cavity and the brain constitute an alternative route by which the meningococci may reach the CNS. To date, the cellular mechanisms involved in nerve infection are not fully understood.

## INTRODUCTION

The olfactory and trigeminal nerves (cranial nerves I and IV, respectively) extend between the nasal cavity and the brain, constituting direct routes to the brain by which pathogens can potentially invade the central nervous system (CNS). The trigeminal nerve has direct contact with the brainstem, while the olfactory nerve terminates in the olfactory bulb. Despite this, infections of the CNS via these routes are rare (reviewed in reference 1). We have previously shown that glial cells, and not macrophages, are the main phagocytes in these nerves (2). Thus, determining how bacteria interact with the glia is important for understanding potential disease progression. While only a discrete number of species are capable of infecting the brain via cranial nerves (1), it remains unknown if this is because these pathogens cannot be cleared by glial cells after phagocytosis. How such bacteria alter the normal biology of glial cells is also unknown.

The Gram-negative facultative diplococcus Neisseria meningitidis is a common cause of sepsis and bacterial meningitis in humans, which often result in a high rate of mortality and morbidity. N. meningitidis is present asymptomatically in the nasopharynx of 4 to 20% of adults (3, 4). The meningococci grow on the surface of mucus-producing epithelial cells, surviving in a nutrient-poor environment with a complex microbiota by expressing key nutrient-capturing and virulence factors (reviewed in reference 5). For unknown reasons, probably relating to lineage-specific virulence factors (6, 7) and potentially to host genetic polymorphisms (8), N. meningitidis can sometimes enter the bloodstream, where the polysaccharide capsule allows survival and replication. Following blood infection, N. meningitidis can penetrate the blood-brain barrier (BBB) or blood-cerebrospinal fluid barrier to infect the meninges; bacterial meningitis is, in turn, the leading cause of CNS infection (9–11). N. meningitidis interacts, via type IV pili, tightly with brain endothelial cells, leading to the formation of microcolonies on the cells (reviewed in references 12–14). This, in turn, leads to activation of intracellular signaling pathways, which results in formation of docking structures. The signaling induced by pathogen-host interactions eventually results in the recruitment of intercellular junction protein and the opening of intercellular junctions, allowing the meningococci to enter the meninges via the paracellular route (reviewed in references 5, 15, 16). N. meningitidis may also move transcellularly across a monolayer of cells with tight junctions, and it can survive intracellularly in microvascular endothelial cells (17); however, definite proof for transcellular passage across the BBB is lacking (16).

While N. meningitidis can be isolated from the bloodstream in the majority of patients, meningococcal sepsis or septic shock only occurs in up to 20% of patients (18, 19), suggesting that nonhematogenous infection path(s) may be important contributors to CNS invasion. N. meningitidis is one of the few species of bacteria that can invade the brain via the nose-to-brain nerve route, as demonstrated for the olfactory nerve (20). We (21) and others (22) have shown that the intranasal trigeminal nerve branch also constitutes a direct route for bacterial invasion of the brainstem in the CNS (reviewed in reference 1); this route is also well known to mediate herpes zoster encephalitis (reviewed in reference 23). We have previously shown that the glia of the trigeminal nerve, trigeminal Schwann cells, readily phagocytose bacteria (24). Infection of phagocytic cells is important in establishing long-term infections (reviewed in reference 23), and other bacteria, including Mycobacterium leprae and Trypanosoma cruzi, can infect and survive within Schwann cells, thereby evading immune destruction and being capable of invading the CNS (reviewed in reference 25).

Prior to this study, it was unknown whether N. meningitidis can infect Schwann cells and whether the bacteria could cause any changes to normal Schwann cell biology. We therefore investigated whether N. meningitidis could infect trigeminal Schwann cells and found that the bacteria readily infected the glia. We found that the infection initiated morphological and protein expression changes in the glia that were consistent with pathology.

## RESULTS

### N. meningitidis C311#3 infection causes nuclear atypia and multinucleation of trigeminal Schwann cells.

**FIG 1:**
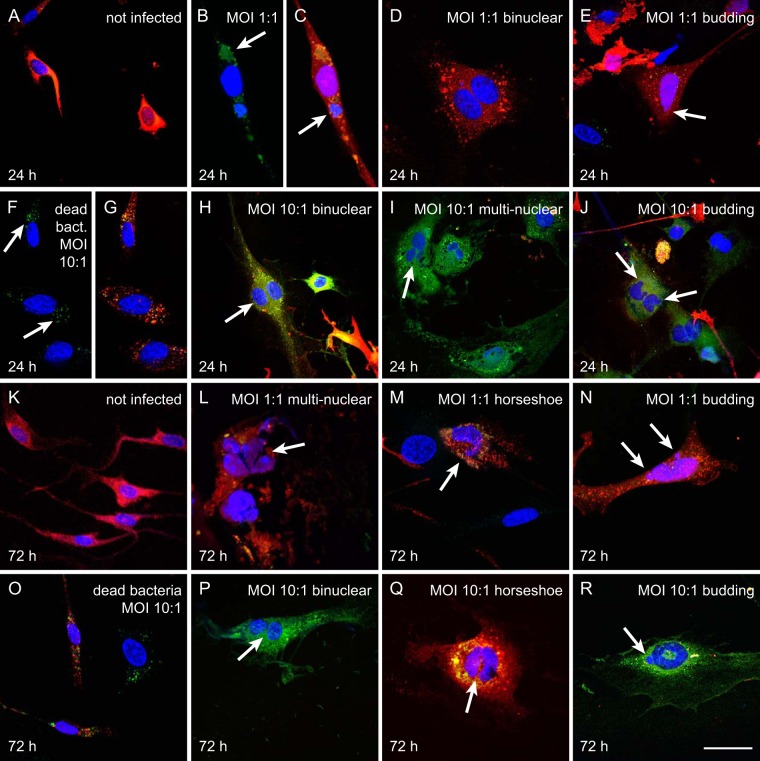
N. meningitidis serogroup B induces nuclear abnormalities in trigeminal Schwann cells. Schwann cells (red) were incubated in the treatments for 24 h (A to J) and 72 h (K to R), with no bacteria (not infected), dead bacteria, or bacteria at an MOI of 1:1 or 10:1 as indicated. Blue fluorescence, 4′,6-diamidino-2-phenylindole (DAPI; nuclear stain); green fluorescence, green fluorescent protein (GFP)-tagged N. meningitidis serogroup B; red fluorescence, DsRed protein in the glial cells (from S100β-DsRed transgenic mice). Shown are typical examples of cells following treatment. At 24 h, (A) control cells (not infected). At an MOI of 1:1, bacteria (arrow) (B) were present in cells with more than one nucleus (arrow) (C and D). (E) Nuclear atypia (budding, indicated by arrow). (F and G) Cells incubated with dead bacteria at an MOI of 10:1 (arrow). (H to J) Cells incubated with bacteria at 10:1 showed bi- and multinucleation (arrows) and budding of nuclei (arrows). At 72 h, (K) control cells not infected with bacteria. At an MOI of 1:1, cells exhibited multinucleation (L), horseshoe nuclei (M), and budding of nuclei (N). (O) Cells incubated with dead bacteria at an MOI of 10:1. (P to R) Cells incubated with bacteria at an MOI of 10:1 showed binucleation, horseshoe nuclei, and budding of nuclei. Bar in panels A to P, 75 μm; bar in panels Q to R, 50 μm.

To determine whether the presence of internalized N. meningitidis affected trigeminal Schwann cells, primary Schwann cells were isolated from the trigeminal nerve of S100β-DsRed transgenic mice, in which the S100β promoter drives the expression of the fluorescent protein DsRed in glial cells (26). The purified Schwann cells express DsRed, enabling easy visualization of the cells as well as verification of cell identity under normal culture conditions. The primary trigeminal Schwann cells were then incubated with N. meningitidis serogroup B (multiplicity of infection [MOI] ratios, 1:1 and 10:1) and examined using immunofluorescence microscopy. After 24 h of incubation, Schwann cells that had not been infected (control cells) were bipolar, and nuclei were of normal oval morphology (Fig. 1A and K) (27).

After 24 h of incubation with N. meningitidis serogroup B (MOI, 1:1), the Schwann cells were infected by the bacteria (green fluorescence; Fig. 1B and C). Some infected cells were binucleated (Fig. 1B to D) with a flattened morphology (Fig. 1D), or had nuclear atypia such as budding nuclei (Fig. 1E). Cells were incubated at a higher MOI (10:1) to determine whether the morphological changes would become more prominent. First, to determine whether the presence of internalized dead bacteria resulted in changes to morphology, cells were incubated with N. meningitidis serogroup B (MOI, 10:1) that had been killed by 4% paraformaldehyde (PFA). Cells incubated with dead bacteria appeared similar to cells that were not incubated with bacteria (Fig. 1F and G). In contrast, cells incubated with live bacteria (MOI, 10:1) exhibited a range of nuclear abnormalities that included binucleation (Fig. 1H), multinucleation (Fig. 1I), and budding of nuclei (Fig. 1J).

We then examined whether the nuclear abnormalities continued to be present with extended incubation time. Control cells that were not infected (Fig. 1K) and cells that were incubated with dead bacteria at an MOI of 10:1 (Fig. 1O) for 72 h maintained their normal bipolar morphology, whereas cells incubated with live bacteria at an MOI of 1:1 or 10:1 exhibited a range of nuclear abnormalities that included multinucleation (Fig. 1L and P), horseshoe-shaped nuclei (Fig. 1M and Q), and budding of nuclei (Fig. 1N and R).

**FIG 2:**
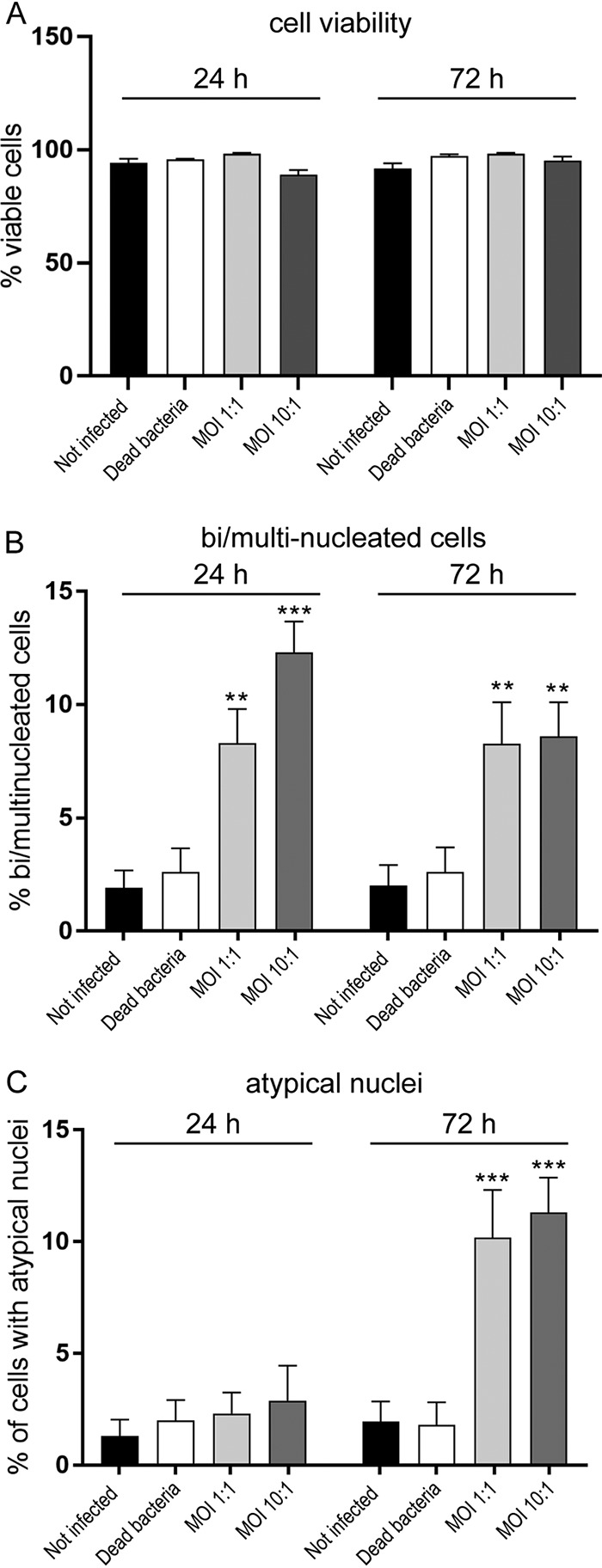
(A) Viability of Schwann cells after infection with N. meningitidis serogroup B with different MOI at 24 and 72 h. Bar graph shows percentage of viable cells after 24 and 72 h, as follows: cells that were not infected, cells incubated with dead N. meningitidis, and cells incubated with live N. meningitidis serogroup B at MOI of 1:1 and 10:1. There were no significant changes in viability of Schwann cells in each treatment group in comparison with noninfected cells. The cells were taken from 5 different animals and pooled; assay was performed in triplicate, with six different FOVs acquired for each condition. One-way analysis of variance (ANOVA) with Tukey’s *post hoc* test. (B and C) Quantification of Schwann cell multinucleation and appearance of atypical nuclei at 24 and 72 h following infection with N. meningitidis serogroup B. Bar graphs show the percentage of infected Schwann cells that exhibited more than one nucleus (B) or atypical nuclei (C) at 24 and 72 h postinfection. Treatment groups were cells that were not infected, cells incubated with dead N. meningitidis, and cells incubated with live N. meningitidis serogroup B at MOI of 1:1 and 10:1. The cells were taken from five different animals, with 3 replicates each with 20 randomly selected fields of view (FOV) comprising 10 to 15 cells/FOV. There was a significant increase in the numbers of bi- and multinucleated cells and cells with atypical nuclei after infection with N. meningitidis at both MOI and time points. Challenging cells with dead bacteria did not change nuclear morphology at either time point in comparison with that of the noninfected cells. **, *P* < 0.01; ***, *P* < 0.001 (compared to noninfected cells; one-way ANOVA and Tukey’s *post hoc* test).

Due to the alterations to nuclear morphology, we assessed whether incubation with the bacteria affected cell viability. The trigeminal Schwann cells were incubated with dead (MOI, 10:1) and live N. meningitidis serogroup B cells (MOI, 1:1 and 10:1) for 24 h and 72 h. The viability assay was performed using Hoechst and Draq7 stains, with Draq7 penetrating dead/permeable cells and thereby enabling determination of the percentage of live/dead cells. The percentages of viable cells after infection (MOI, 1:1 and 10:1) were not significantly different from those of cells that were not infected or cells incubated with dead bacteria (MOI, 10:1) (Fig. 2A).

We next quantified the percentage of the Schwann cells that (i) were bi- or multinucleated and (ii) exhibited atypical nuclei after 24 and 72 h. Nuclear atypia were defined as nuclei with an abnormal appearance, including a horseshoe-shaped nucleus, a circular nucleus with a hole in the middle, or budding (rounded protrusions emanating from the larger nucleus). Control cells that were not infected exhibited a low level (1 to 2%) of multinucleation or atypical nuclei at both 24 and 72 h (Fig. 2B and C). Similarly, cells incubated with dead bacteria (MOI, 10:1) exhibited low levels of bi- and multinucleation (2.6%) or atypical nuclei (1.8%) that were not significantly different than those of cells that were not infected. In contrast, when the cells were infected with live bacteria at an MOI of 1:1 or 10:1 for 24 h, the percentage of cells that were bi- or multinucleated was significantly higher (MOI = 1:1, 8%; MOI = 10:1, 12%) than those of control cells that were not infected (Fig. 2B). While cells incubated with live bacteria exhibited some nuclear atypia at 24 h (Fig. 1E), the levels were not significantly different from those of the control (Fig. 2C). With 72 h of incubation, cells incubated with dead bacteria (MOI, 10:1) continued to exhibit low levels of bi- and multinucleation (2%) and atypical nuclei (2.6%), which were not significantly different from levels in cells that were not infected. In contrast, cells incubated with live bacteria for 72 h exhibited significantly higher levels of bi- and multinucleation (8.2% and 8.6%; MOI = 1:1 and 10:1, respectively) than control cells (Fig. 2B). In addition, the percentages of atypical nuclei increased to 10.2% and 11.3% (MOI = 1:1 and 10:1, respectively) which were significantly higher than those of control cells that were not infected (Fig. 2C). Thus, while bi- and multinucleation of Schwann cells induced by N. meningitidis occurs rapidly, the induction of atypical nuclei is slower.

**FIG 3:**
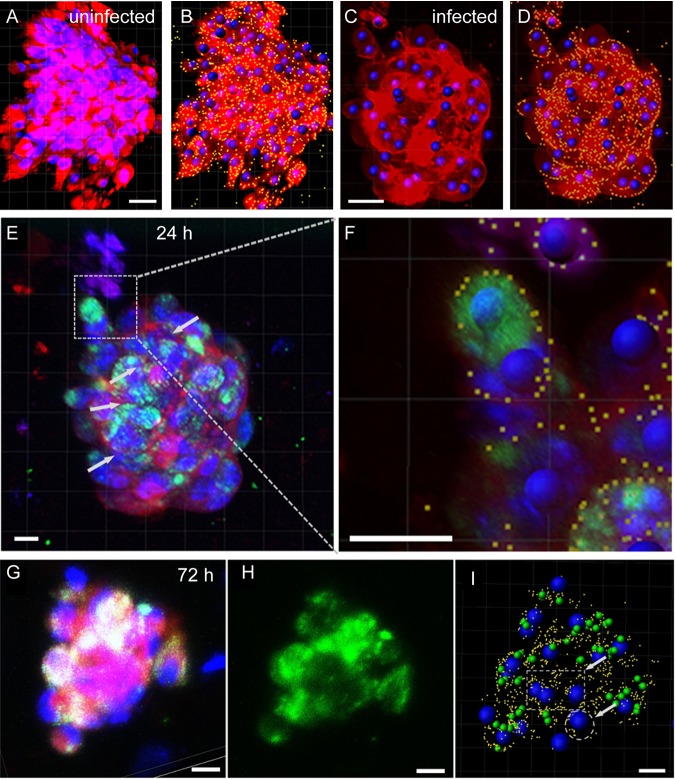
Trigeminal Schwann cells infected with N. meningitidis in 3D cell culture become multinucleated. (A) Trigeminal Schwann cells (not infected) in a 3D spheroid stained with Hoechst (nucleus, blue) and CellMask (cell membrane, red) stains. (B) Nuclei were recognized by Hoechst stain and size, whereas cell membrane boundaries were recognized by intensity (yellow dots). Computerized image analysis was then used to localize nuclei within each cell. (C to F) Trigeminal Schwann cells in 3D cell culture infected with N. meningitidis for 24 h. (C) Nuclei were recognized by Hoechst staining and size. (D) Cell membrane boundaries were recognized by intensity of CellMask staining. Fluorescence of bacteria is not shown in panels C and D, but is shown in panel E. (E) N. meningitidis infects the Schwann cells within the 3D culture; green fluorescence corresponds to GFP-expressing C311#3. Computerized analysis localizing nuclei within each cell showed that 24 h after infection with N. meningitidis (MOI, 10:1), multinucleated Schwann cells were present within the spheroid. (F) Enlargement of a multinucleated Schwann cell; at least three nuclei are present within the cell border. (G to I) 3D-cultured trigeminal Schwann cells infected with N. meningitidis for 72 h. Blue, nuclei (Hoechst); red, cell membranes (CellMask); green, GFP-expressing C311#3. (G) N. meningitidis infects the Schwann cells within the 3D culture; green fluorescence corresponds to GFP-expressing C311#3 (H). (I) Computerized analysis revealed several multinucleated cells (dotted circles/square) within the culture. Blue, nuclei; yellow, cell boundaries; green, N. meningitidis. For each time point, at least *n* = 5 to 10 spheroids were generated. Bar in panels A and B, 30 μm; bar in panels C to I, 10 μm. Images were captured by Nikon AR1+ and image analysis was done with Imaris 9.0.

Three-dimensional (3D) cell cultures more closely model *in vivo* cell relationships, as cell interactions are more complex, with local autocrine and paracrine signaling. Thus, the cell responses in 3D cultures may differ from those of cells in two-dimensional (2D) cultures (28, 29). 3D cultures of neural cells are thus considered more appropriate for modeling nervous system infections *in vitro* than 2D cultures (30). We therefore examined the response of trigeminal Schwann cells to N. meningitidis in 3D cell cultures. We have developed a novel method for 3D cell culture termed the naked liquid (NLM) marble system, in which cells spontaneously and rapidly form 3D spheroid structures. The cells exhibit similar cell-cell interactions to those *in vivo*, and, therefore, this culture system mimics the *in vivo* milieu better than 2D cell culture (31). Suspensions of single cells were seeded into naked liquid marbles and incubated overnight to allow the cells to form 3D spheroids, as previously described (31). The 3D cultures were then infected with N. meningitidis (MOI, 10:1) for 24 h and 72 h. Following incubation with bacteria, the response of trigeminal Schwann cells within the spheroids to N. meningitidis was examined using confocal microscopy. Due to the close contact of cells within the 3D spheroids, we were not able to use confocal microscopy analysis to determine cell boundaries in all situations, so quantification of the number of bi- and multinucleated cells (MNCs) could not be performed. Nevertheless, image analysis using Imaris software, which detected cell boundaries defined by CellMask stain in some cells, showed that multinucleated cells were present after 24 h (Fig. 3C to F) and 72 h (Fig. 3G to I) of incubation with bacteria. In contrast, multinucleated cells were largely absent from the uninfected spheroids (Fig. 3A and B).

### Infection with N. meningitidis causes alteration in gliomagenesis markers.

**FIG 4:**
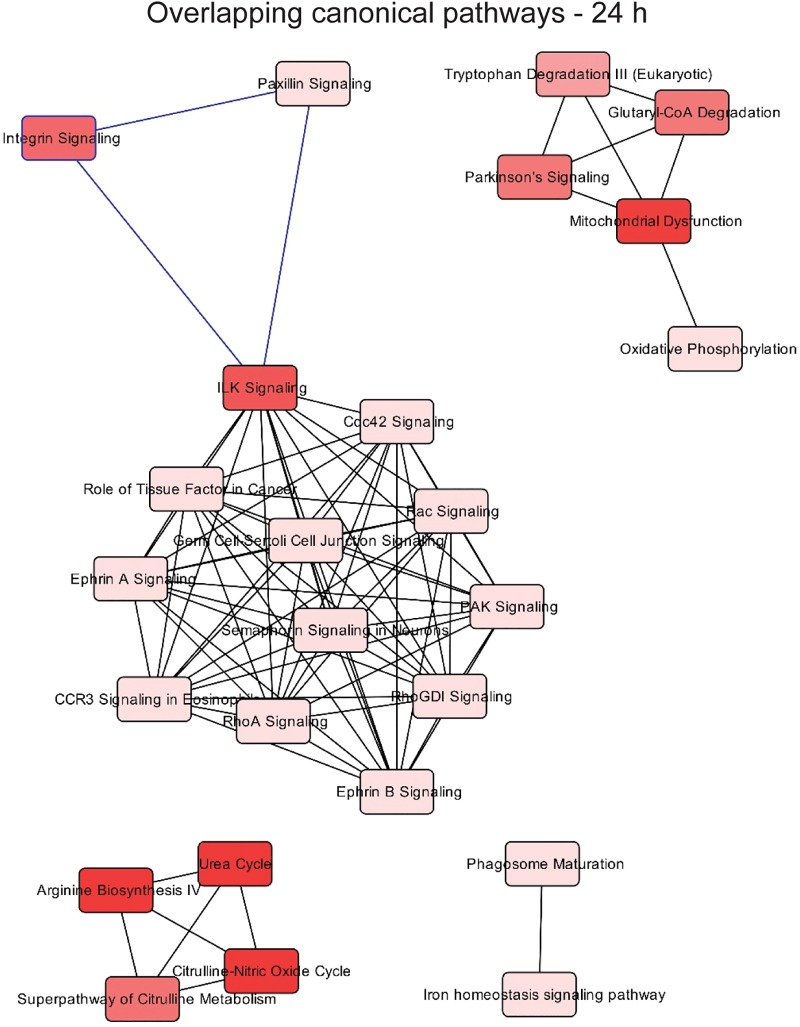
Network associated with the top 25 affected pathways with differentially abundant proteins after infection for 24 h, according to Ingenuity Pathway Analysis. SWATH-MS proteomics was performed on *n* = 3 × 100,000 cells for each incubation time.

**FIG 5:**
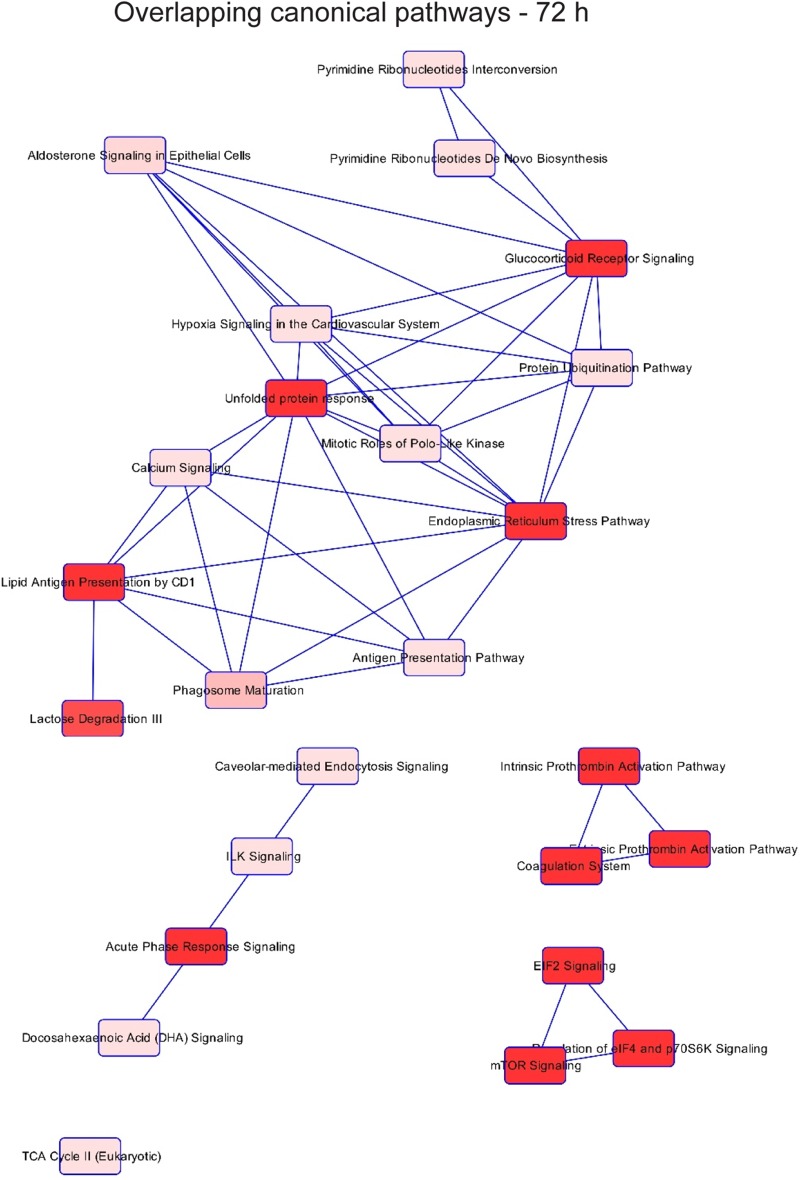
Network associated with the top 25 affected pathways with differentially abundant proteins after infection for 72 h, according to IPA.

**FIG 6:**
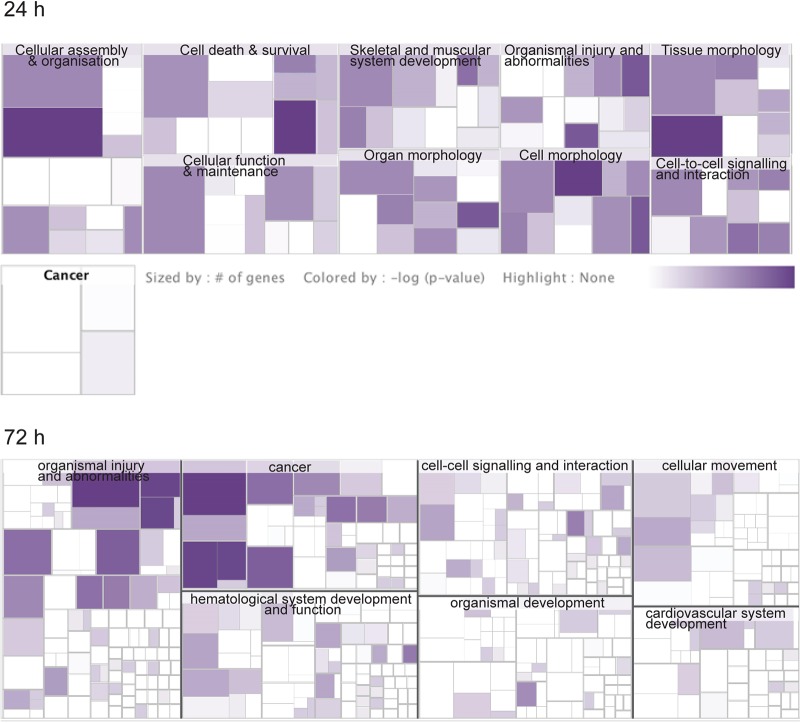
Expression variability of specific proteins involved in diseases following infection with N. meningitidis C311#3 at (top) 24 h and (bottom) 72 h, with the number of proteins affected (indicated by size) and the *P* value (indicated by color). The most highly affected diseases are shown in the main panels; cancer-related protein changes are displayed at 24 h to demonstrate that they were minimally affected.

Multinucleation and abnormal nuclei are associated with various pathologies. To gain insight into which potential pathologies may be associated with N. meningitidis infection of Schwann cells, we performed quantitative sequential window acquisition of all theoretical mass spectra (SWATH-MS) proteomics to determine which proteins had altered expression. Of 929 proteins examined (496 across 24 h and 433 across 72 h of infection), 185 proteins showed statistically significant changes in their abundance (adjusted *P* value, <0.05) following infection. Pathway analysis was then performed using Ingenuity Pathway Analysis (IPA). While numerous canonical pathways associated with several diseases (see Fig. 6, top) were affected at 24 h (Fig. 4), by 72 h after infection, apart from general organismal injury and abnormalities, a number of intercellular signaling, cell-cell interaction, and cellular movement pathways were affected (Fig. 5), with cancer most strongly correlating with the alterations in protein expression at 72 h (Fig. 6, bottom). In contrast, at 24 h, cancer-associated proteins were minimally affected (Fig. 6, top). The SWATH-MS and IPA analyses identifying the strong cancer pathway alterations at 72 h are consistent with the histological analysis, which demonstrated nuclear atypia were most prominent at 72 h (Fig. 1 and 2). Key cancer-related and cell signaling/movement proteins affected are listed in Table 1. The full list of SWATH-MS proteomics data is shown in Table S1.

**TABLE 1:** Alteration in levels of some proteins involved in cancer after infection with N. meningitidis in trigeminal Schwann cells^*a*^

Protein	Description/function^*b*^	Change in protein level after incubation^*c*^		Incubation time (h)
		log_2_FC	FC (*P* value)	
Phosphoglycerate mutase 1 (PGAM1)	Putative tumor suppressor, inhibits tumor growth and metastasis in several cancers. PGAM1 is downregulated in glioma cells (74). Thus, downregulation may be associated with cancer.	−0.54	↓ 1.5 (<0.001)	24
Plasminogen activator inhibitor 2 (PAI2)	Cytoprotective Rb-binding protein; protects Rb from cleavage by calpain, causing upregulation of Rb, which promotes cell survival in cancer (59). Thus, upregulation of PAI2 may be associated with cancer.	1.05	↑ 2 (<0.001)	24
Profilin 1 (PROF1)	Putative tumor suppressor which inhibits tumor cell growth and metastasis in several cancers. Downregulation of profilin 1 reduces tumor suppression and causes tumorigenesis (58). Thus, downregulation may be associated with cancer.	−0.23	↓ 1.2 (<0.001)	24
Vacuolar protein sorting-associated protein 35 (VPs35)	Essential retromer subunit for the *wnt* signaling pathway. Loss of VPs35 results in inhibited *wnt* signaling, which is seen in many cancers (75). Thus, downregulation may be associated with cancer.	−1.86	↓ 3.7 (<0.001)	24
Annexin A1 (ANXA1)	Adhesion protein, downregulates Cox-2 expression. Loss of annexin 1 leads to the overexpression of Cox-2, which is seen in cancer (60). Thus, downregulation may be associated with cancer.	−0.18	↓ 1.1 (<0.001)	72
Fibronectin C (FinC)	Cell adhesion protein. Loss of fibronectin leads to loss of contact inhibition of cell movement and proliferation, promoting invasion of neighboring tissues and metastasis to remote organs, especially in head and neck cancers (76). Thus, downregulation may be associated with cancer.	−0.78	↓ 1.7 (<0.001)	72
Pigment epithelium-derived factor (PEDF)	A secreted glycoprotein that is widely expressed by multiple organs (77); a critical factor in controlling stemness and tumor progression of glioma stem cells. Promotes cell migration and tumor metastasis through an interaction with the laminin receptor (78). Thus, upregulation may be associated with cancer.	0.40	↑ 1.3 (<0.001)	72
Serum amyloid P component (SAP)	A member of the lectin fold superfamily and the pentraxin serum protein family (79, 80). Correlation between SAP level of expression and carcinoma and the severity of the disease has been demonstrated (81). Therefore, upregulation may be associated with cancer.	0.33	↑ 1.3 (<0.001)	72

a As determined by SWATH-MS proteomics.

b Rb, retinoblastoma.

c Arrows indicate increase (↑) or decrease (↓) in FC. log_2_FC, log_2_ fold change; FC, fold change.

## DISCUSSION

**FIG 7:**
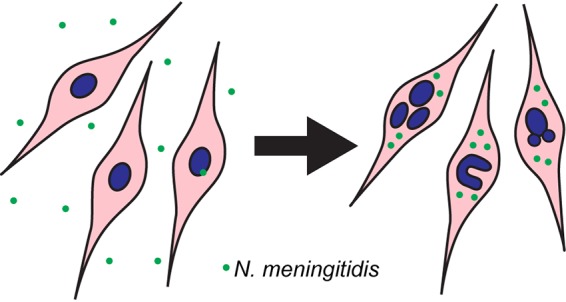
Schematic summary. Neisseria meningitidis infects trigeminal Schwann cells, resulting in the formation of multinuclear cells and/or cells with budding nuclei or horseshoe nuclei. These atypical nuclei are present in various pathologies, including glioma.

A small number of bacterial species are thought to be capable of invading the CNS via the cranial nerves that extend between the nasal cavity and the brain, the trigeminal and olfactory nerves. We hypothesized that such bacteria may be capable of infecting the glial cells of these nerves and potentially modulating the biology of the glia. We investigated here how one such species, Neisseria meningitidis, affected trigeminal nerve Schwann cells. Our results show that N. meningitidis infected the cells at low and high MOI, resulting in the formation of multinucleated cells and the appearance of atypical nuclei (Fig. 7). The nuclear morphological changes were dependent on the presence of live bacteria, as internalized dead bacteria did not result in nuclear changes. The infection of trigeminal Schwann cells may constitute a mechanism by which N. meningitidis can invade the trigeminal nerve and subsequently reach the CNS.

To study the cellular responses to the infection in a setting resembling the *in vivo* environment, we also infected 3D cultures of trigeminal Schwann cells with N. meningitidis. Our results demonstrated that N. meningitidis infected the cells cultured in the 3D format and induced multinucleation in some cells. Here, it is important to consider the reaction of cells in 3D cultures compared to that in 2D cultures. While 2D cultures provide clear visualization of cell interactions, 3D models can better reflect *in vivo* cell interactions (28–30, 32, 33). In 2D cultures, all cells are exposed to bacteria, whereas in 3D cultures it is initially only the cells on the exterior of the 3D spheroid that are exposed. For the external cells, the potential trophic and cell-cell contact with interior cells may confer resistance to the changes induced by bacteria. Multinucleated cells were detected in the 3D cultures with N. meningitidis, similarly to the 2D cultures, suggesting that the morphological changes are consistent across the different culture formats. We could not image the 3D cultures at a sufficiently high resolution to determine whether or not atypical nuclei were found in the 3D-cultured cells.

Multinucleation and nuclear atypia are key features of cells in glioma tumors (34), particularly of one variant termed giant cell glioblastoma (35). Multinucleated giant cells and cells with atypical nuclei can be found in malignant and nonmalignant schwannomas (Schwann cell tumors), of which the vast majority are nonmalignant (36–42). The presence of multinucleated cells (MNCs) in itself may not be indicative of cancer. MNCs are formed as cells react to foreign bodies and to viral infections (reviewed in references 43, 44). However, MNCs in combination with nuclear atypia in glial cells are a key characteristic of glioma tumors (45), including schwannomas (36–42), with the degree of nuclear atypia correlating with tumor staging (46, 47). The origin of MNCs in glioma is not yet understood. It has been shown that the cells remain in the early mitotic phase, which involves dysregulation of the P53 protein (48); however, the mechanisms and, in particular, the roles of external/environmental factors are unknown. Some bacteria are known to cause the formation of multinucleated cells in other cell types, for example, in mycobacteria, such as Mycobacterium tuberculosis and Chlamydia spp., inhibiting cell division (reviewed by references 43, 49), and in Burkholderia pseudomallei, causing cell-cell fusion (50, 51). Out of these, M. tuberculosis (52, 53) and Chlamydia pneumoniae (54, 55) have been linked to lung cancer, and Chlamydia trachomatis has been linked to cervical cancer (56, 57).

As multinucleation and atypical nuclei are associated with a range of pathologies, we performed SWATH-MS proteomics to identify which biological and disease pathways were altered following infection with N. meningitidis. Comparison of the trigeminal Schwann cell proteome in infected cells and control cells showed that N. meningitidis altered the expression of several pathways, including dysregulation of cancer-related proteins. N. meningitidis infection caused downregulation of proteins responsible for tumor suppression, such as profilin 1, and upregulated the antiapoptotic protein serpin B2 (58, 59). The proteomics data also suggest that N. meningitidis increases cellular proliferation capacity and cellular invasiveness by downregulation of annexin1 (Cox-2 inhibitor) and fibronectin C, respectively (60, 61). Other pathways, such as the endoplasmic reticulum stress pathway, the unfolded protein response pathway (62), and the EIF2 pathway (63), were also affected.

In this study, we used mouse trigeminal glia. Humans are the only natural host of N. meningitidis; however, intranasal inoculation of mice is often used to model N. meningitidis infection in the laboratory (20, 64, 65), and infection of primary mouse cells, including astrocytes and microglia, has previously been demonstrated (66). Ideally, however, the glial responses to N. meningitidis that we report here should be confirmed in human trigeminal Schwann cells. Unfortunately, primary trigeminal Schwann cells cannot be obtained from humans due to the anatomy of the trigeminal nerve and are not commercially available to date.

In summary, the results of the current study suggest that N. meningitidis can initiate cellular and molecular changes in trigeminal Schwann cells. The cellular changes include (i) the formation of multinucleated cells, (ii) the induction of nuclear atypia, and (iii) alterations in the levels of proteins responsible for cellular hemostasis and proliferation. While these cellular changes are associated with a range of pathologies, the proteomic pathway analysis indicated, interestingly, that cancer-related changes were predominately affected.

## MATERIALS AND METHODS

### Cell culture.

Primary trigeminal Schwann cells were isolated from S100β-DsRed transgenic mice, in which the S100β promoter drives the expression of the fluorescent protein DsRed in glial cells (26), according to our previously published method (24). Briefly, S100β-DsRed postnatal day 7 (P7) pups were decapitated, followed by dissection of the trigeminal nerve immediately adjacent (anterior) to the trigeminal ganglia. Explants of the tissue were added to 24-well plates previously coated with Matrigel (1:10; BD Bioscience) in small droplets of glial medium (Dulbecco’s modified Eagle medium containing 10% fetal bovine serum, G5 supplement [Gibco], gentamicin at 50 μg/ml [Gibco], and l-glutamine at 200 μM). The identity of Schwann cells was verified using expression of DsRed and immunohistochemistry as we described previously (24, 27). All procedures were carried out with the approval of the Griffith University Animal Ethics Committee under the guidelines of the Australian Commonwealth Office of the Gene Technology Regulator.

### Bacterial strains and media.

The meningococcal strain used in this study is C311#3-GFP (67) which is a C311#3 (serogroup B) strain transformed with the green fluorescent protein (GFP) expressing plasmid pCmGFP (68). C311#3-GFP cells were grown on brain heart infusion (BHI), (1%) agar (10%) (both from Oxoid), and Levinthal’s Base medium supplemented with chloramphenicol (5 μg/ml) at 37°C with 5% CO_2_ for 16 to 18 h. Bacterial cultures were incubated in BHI broth (at 37°C, with shaking) for 4 h, after which the optical density of the cultures was adjusted to 10^9^ meningococcal cells per ml and used to infect Schwann cells.

### Infection of Schwann cells with N. meningitidis, microscopy, and quantification of nuclear abnormalities.

To determine the effects of N. meningitidis infection on the morphology of trigeminal Schwann cells, with particular focus on nuclei, the cells were imaged using confocal microscopy after infection with fluorescence-labeled bacteria. Trigeminal Schwann cells from explant cultures were seeded and cultured at a density of 5,000 cells per well in glass-bottomed 8-well chambers (Sarstedt) in glial medium. Twelve h after seeding, cells were infected with GFP-expressing N. meningitidis (C311#3) at a multiplicity of infection (MOI) of 10:1 in antibiotic-free glial medium for 90 min, after which the medium was removed, and the cells were washed 3 times with gentamicin-containing medium and then incubated in medium with gentamicin for 24 h and 72 h (61). We also studied the morphology of nuclei after uptake of dead bacteria. GFP-tagged N. meningitidis bacteria were killed by incubation with 4% PFA for 10 min. Trigeminal Schwann cells were then incubated with the dead bacteria at an MOI of 10:1 for 24 and 72 h, with the dead bacteria washed off at 90 min as per the protocol for live bacteria. Following incubation, cells were rinsed in 1× Hanks’ balanced salt solution and were fixed for 20 min in 4% paraformaldehyde (PFA) in Dulbecco’s phosphate-buffered saline (DPBS) and then rinsed in DPBS 3 times for 5 min. Subsequently, nuclei were stained using 4′,6-diamidino-2-phenylindole (DAPI) for 5 min at room temperature. Cells were imaged using confocal microscopy (FluoView FV1000 microscope; Olympus). We manually quantified the percentage of cells that (i) were bi/multinucleated or (ii) showed sign of nuclear atypia by counting cells with more than one nucleus or with atypical nucleus using a tally counter. Nuclear atypia were defined as nuclei with an abnormal appearance, including a horseshoe-shaped nucleus, a circular nucleus with a hole in the middle, or budding (rounded protrusions emanating from the larger nucleus). DsRed cells colocalized with DAPI staining were analyzed in 20 randomly selected fields of view (FOV) comprised of 10 to 15 cells/FOV. These experiments were repeated three times (biological replicates). Measurements were tested for statistical significance using one-way analysis of variance (ANOVA) with Tukey’s *post hoc* analysis.

### Viability (live/dead cell) assay.

A live/dead cell assay was performed on trigeminal Schwann cells challenged with live and dead bacteria after 24 h and 72 h. We used Hoechst (1:1,000) and Draq7 (1:500) nuclear stains on unfixed cells after each time point under two different conditions. While Hoechst was used for staining all nuclei, Draq7 was used to stain only dead/permeable nuclei. All images were acquired using a Nikon Eclipse Ti2 widefield microscope for Hoechst (405 nm) and Draq7 (647 nm). The cells were obtained from 5 different animals and pooled; the assay was performed in triplicate, with six different FOVs acquired for each condition. Viability % was measured using the following formula:

$$\text{viability }\%=\frac{\text{total no. of Hoechst stained cells }-\text{ total no. of Draq7-stained cells}}{\text{total no. of Hoechst stained cells}}\text{ × 100}$$

### Infection of trigeminal Schwann cells cultured in three dimensions with N. meningitidis.

To determine whether N. meningitidis infected and caused nuclear changes in 3D-cultured trigeminal Schwann cells, we generated naked liquid marbles (NLMs) containing trigeminal Schwann cells. Our laboratory has developed the NLM platform in which droplets of cell culture medium are incubated on a superhydrophobic coating (31, 69). Inside the NLMs, cells are free to interact, forming multiple 3D spheroids that are uniform in size and shape in less than 24 h. A micropipette was used to dispense the required volume of Schwann cell medium containing 385 cell/μl to form NLMs with a volume of 20 μl (7,700 cells per NLM). The cells were incubated overnight in 5% CO_2_ in air at 37°C. Following incubation, the cell spheroids were infected with GFP-tagged N. meningitidis (MOI, 10:1) for 24 h and 72 h. Following the infection, the spheroids were rinsed in 1× Hanks’ balanced salt solution (HBSS) and fixed for 20 min in 4% PFA in DPBS. After fixation, the cells were rinsed in DPBS 3 times for 5 min. Hoechst was added to stain nuclei, and the spheroids were subsequently visualized by confocal microscopy (AR1+ laser scanning confocal microscope; Nikon). Image analysis of cells within the 3D spheroids was conducted using Imaris 9.0 software to determine multinucleation.

### SWATH-MS proteomics.

To study the changes in the protein expression and proteome alterations in the host mammalian cells after infection with N. meningitidis, sequential window acquisition of all theoretical mass spectra (SWATH-MS) proteomics was performed following the infection in trigeminal Schwann cells. Schwann cells were incubated with C311#3 (MOI, 10:1 for 24 h and 72 h), and then washed with cold phosphate-buffered saline (PBS). Cells were harvested (*n* = 3 × 100,000 cells for each incubation time), lysed in 250 μl 6 M guanidine-HCl, 50 mM Tris-HCl (pH 8), and 10 mM dithiothreitol (DTT), and incubated at 30°C for 30 min. Cysteines were alkylated by addition of acrylamide to a final concentration of 25 mM and incubation at 30°C for 30 min. Proteins were precipitated by addition of 1 ml of 1:1 methanol/acetone and incubation overnight at −20°C. After centrifugation at 18,000 relative centrifugal force (rcf) for 10 min and removal of the supernatant, the protein pellet was resuspended in 100 μl of 50 mM Tris-HCl (pH 8) with 1 μg of trypsin and incubated overnight at 37°C. Tryptically digested peptides were desalted with C_18_ ZipTips (Millipore). Mass spectrometry was performed by liquid chromatography-tandem mass spectrometry (LC-MS/MS) using a Prominence nanoLC system (Shimadzu) and TripleTOF 5600 instrument with a NanoSpray III interface (Sciex) essentially as described previously (70). For data-dependent acquisition analysis, ∼2 μg of desalted peptides was separated on an Everest reversed-phase C_18_ column (Vydac). Peptides were separated with buffer A (1% acetonitrile and 0.1% formic acid) and buffer B (80% acetonitrile with 0.1% formic acid) with a gradient of 10 to 60% buffer B over 45 min. An MS-time of flight (TOF) scan was performed from *m/z* of 350 to 1,800 for 0.5 s, followed by data-dependent acquisition of MS/MS of the top 20 peptides from *m/z* 40 to 1,800 for 0.05 s per spectrum, with automated capillary electrophoresis (CE) selection. For data-independent acquisition of SWATH-MS, ∼0.5 μg of desalted peptides of three biological replicates was separated using identical LC parameters as for data-dependent acquisition. An MS-TOF scan was performed from *m/z* 350 to 1,800 for 0.05 s, followed by high-sensitivity information-independent acquisition with 26 *m/z* isolation windows with 1 *m/z* window overlap each for 0.1 s across an *m/z* range of 400 to 1,250. The collision energy was captured and assigned by Analyst software (Sciex) based on *m/z* window ranges. The proteins were identified from data-dependent acquisition data using ProteinPilot 5.1 (Sciex), searching against a database of all predicted mouse proteins, with the following settings: sample type, identification; cysteine alkylation, acrylamide; instrument, TripleTOF 5600; species, none; ID focus, biological modifications; enzyme, trypsin; search effort, thorough ID. The results from ProteinPilot were used as an ion library to measure the abundance of peptides and proteins using PeakView 2.1 (Sciex), with the following settings: shared peptides, allowed; peptide confidence threshold, 99%; false discovery rate, 1%; XIC extraction window, 6 min; XIC width, 75 ppm. False-discovery rate analysis was performed on all searches. ProteinPilot search results were used as ion libraries for SWATH analyses. The abundance of proteins was measured automatically using PeakView (Sciex) with standard settings, and the comparison relative abundance of protein was performed based on protein intensities. Statistical analyses were performed as previously described (71), using ReformatMS (72) and MSstats (2.4) (73). Proteins with adjusted *P* values of <0.05 were considered significant. All experiments were done in three biological replicates.

### Pathway analysis.

Pathway analysis was performed for proteins with differential abundance between infected and noninfected groups using Ingenuity Pathway Analysis (IPA; Qiagen Bioinformatics). IPA is a Web-based software application for the analysis, integration, and interpretation of the data derived from ‘omics analysis, including proteomics. UniProt accession number, log_2_ fold change (log_2_FC), and adjusted *P* values were uploaded for the two time points for log_2_FC; 0.05 for the adjusted *P* value was used to define significant differential abundance.

Overlapping networks among the top 25 canonical pathways detected as significant were built for each time point. Each node corresponds to a canonical pathway detected as significant, and links between nodes indicate that at least 1 molecule is shared between two pathways. Color brightness of nodes indicates the significance of the pathway; the darker the color, the more significant the pathway.

Tree maps were used to represent the biological impact resulting from the differentially abundant proteins. Each major box represents top-level biological functions or diseases, within which each individual rectangle is a subfunction related to the top-level function. The size of a subrectangle is proportional to the number of differentially abundant proteins, and its darkness is proportional to its significance.

## Supplementary Material

Supplemental file 1
